# Bite Force of 3-6-Year-Old Children After Unilateral Extraction of Primary Teeth

**Published:** 2018-01

**Authors:** Alireza Heydari, Yahya Baradaran Nakhjavani, Elnaz Askari Anaraki, Siavash Arvan, Maryam Shafizadeh

**Affiliations:** 1 Assistant Professor, Dental Research Center, Dentistry Research Institute, Tehran University of Medical Sciences, Tehran, Iran; Department of Pediatric Dentistry, School of Dentistry, Tehran University of Medical Sciences, Tehran, Iran; 2 Associate Professor, Department of Pediatric Dentistry, School of Dentistry, Tehran University of Medical Sciences, Tehran, Iran; 3 Postgraduate Student, Department of Pediatric Dentistry, School of Dentistry, Tehran University of Medical Sciences, Tehran, Iran; 4 Dentist, Private Practice, Tehran, Iran

**Keywords:** Bite Force, Tooth, Deciduous, Tooth Extraction

## Abstract

**Objectives:**

This study aimed to assess the bite force of 3-6-year-old children in primary dentition period after unilateral extraction of a primary first molar (D) and its correlation with the height, weight, gender, type of occlusion, and temporomandibular disorders (TMDs).

**Materials and Methods:**

Twenty children between the ages of 3 and 6 years with a unilaterally extracted D comprised our case group, and 29 age-matched children with no extracted teeth comprised the control group. The maximum bite force at the site of posterior teeth was measured using a bite force measuring device with a 0.2-mm thickness and 3-cm diameter, attached to a strain-gage sensor. Each child bit the sensor with maximum force for 3 seconds, and this was repeated three times at 10-minute intervals. The mean value was calculated. Data were analyzed using SPSS 18 software program via generalized estimating equation (GEE).

**Results:**

the bite force on the side of extraction was significantly lower than that on the contralateral side (P<0.05). Also, the bite force was significantly correlated with the height, gender, and age, but the correlations between the bite force and weight, type of occlusion and side of the jaw were not significant (P>0.05).

**Conclusions:**

Extraction of primary first molars decreases the bite force on the respective side of the jaw.

## INTRODUCTION

Bite force is regulated by the neural, muscular, skeletal, and dental systems [1]. Thus, impairment of these systems directly affects the pattern of chewing and bite force [1]. The strength of masticatory muscles determines the bite force. Mastication may be enhanced by an increase in the bite force [2]. Evidence shows that a significant association exists between chewing, strength of masticatory muscles, number of posterior teeth participating in occlusion and volume and thickness of the alveolar process [3]. Increased bite force causes tension in the alveolar process. If this force is within the normal range, it will physiologically regulate bone maturation. Higher loads cause hypertrophy of alveolar processes [3,4]. Thus, the bite force is directly correlated with the efficiency of the masticatory system [2]. Knowledge about the bite force plays an important role in the success of restorative and prosthetic treatments. Also, such information is required for the manufacturers to produce restorative materials with a sufficient durability under masticatory forces [5]. Bite force increases with age. It remains constant between the ages of 20 and 40 years, and decreases afterwards [6]. Tooth extraction decreases the bite force [7]. Anatomical and physiological parameters such as craniofacial morphology, age, gender, periodontal support, temporomandibular disorders (TMDs), pain in the temporomandibular joint (TMJ), and dental status also affect the bite force [6]. In addition, accurate measurement of the bite force depends on the mechanical and electronic properties of the measuring device [5]. Malocclusion and dental caries affect the bite force as well [5,7]. Evidence shows a wide variability in the bite force, which may be due to the facial structure, strength of the head and neck muscles, and gender [8,9]. The site of measurement of the bite force and the vertical distance between the two jaws can also influence the bite force [10]. Bite force affects the performance of muscles and development of the masticatory system. It becomes progressively greater with an increased need for mastication, number of teeth in occlusal contact, number of erupted teeth, development of the dental system, and increased weight and height [11], while it decreases by deterioration of dentition or pain in the TMJ [12,13]. Bite force measurement is extensively performed to understand the mechanical principles of chewing and to assess the therapeutic effects of dental prostheses [14]. Each individual has equal bite forces on the two sides of the jaw. Extraction of teeth on one side causes an imbalance in the bite force. The prevalence of dental caries has increased in primary dentition, which may lead to tooth loss [15], and since no previous study is available on the bite force of the Iranian children or on the assessment of the effect of physiological factors on the bite force, this study aimed to assess the bite force of 3-6-year-old children in primary dentition period with an extracted primary first molar (D) on one side of the jaw. The associations between the bite force and height, weight, age, gender, type of occlusion and TMJ problems were also assessed.

## MATERIALS AND METHODS

This descriptive cross-sectional study was conducted on 49 children between the ages of 3 and 6 years, selected from the kindergartens of district 6 of Tehran using convenience sampling. The study protocol was approved by the ethics committee of Tehran University of Medical Sciences (Ethical code: 5081). The minimum required sample size was calculated to be 18 children in each group based on previous studies [16,17], but for more accuracy, the samples were increased to 20 children in each group. The case group included 20 children between the ages of 3 and 6 years (before the eruption of permanent first molars) with an extracted D on one side of the jaw (extracted within six months ago). The control group included 29 children between the ages of 3 and 6 years with all the primary teeth present in the mouth.

The age, sex, height, weight, type of occlusion and symptoms of TMD were recorded. An accurate dental examination was performed, and children with spontaneous pain, periodontal diseases, facial asymmetry, a history of trauma, maxillofacial surgery, chewing disorder, or occlusal interference were excluded. The parents signed written informed consent forms after they were briefed about the study’s aim and method. The maximum bite force was measured using a bite force measuring device (Shahid Beheshti University of Medical Sciences (SBMU), Tehran, Iran) with a 0.2-mm thickness and a 3-cm diameter, attached to a strain-gage sensor. The maximum bite force was measured with the child in a seated position and the occlusal plane parallel to the horizon at the site of primary first and second molars. Each child bit on the sensor with maximum force for 3 seconds, and this process was repeated three times at 10-minute intervals. The mean of the three values was calculated. The sensor was covered with disposable covers for each patient.

### Characteristics of the bite force measuring device:

**Fig. 1:**
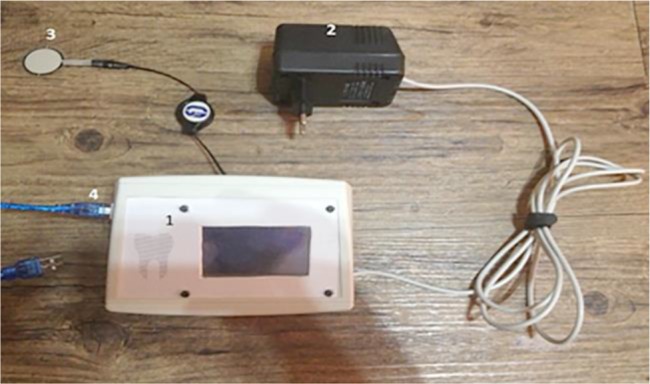
Components of the bite force measuring device. (1) Body; (2) Power supply; (3) Sensor; (4) PC connector

**Fig. 2:**
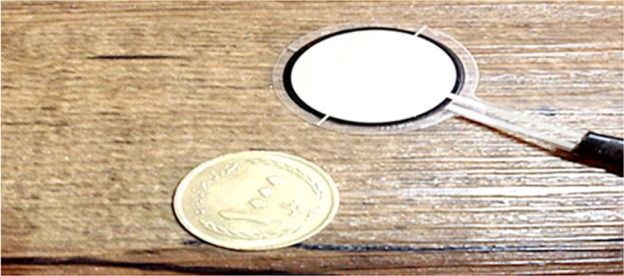
Flexible sensor with a scale to understand the real size

Figure 1 shows the components of the bite force measuring device. A FlexiForce sensor (A401, Tekscan Inc., South Boston, MA, USA) was used. The cross-sectional area of the sensor to which the force is applied does not affect the value displayed by the device. Table 1 shows the physical properties of the sensor. This sensor served both as a strain-gage (measuring the flexural loads applied to the sensor) and a load cell (measuring the vertical loads applied to the sensor). As a result, the operator could measure all the loads applied by teeth to the sensor during mastication and display the outcome of the designed circuit as a numerical value in Newton (N). The range of loads and unsaturation of the sensor during load application could be adjusted by changing the input voltage. Figure 2 shows that at lower input voltages, saturation of the sensor would occur at higher loads, and the range of load measurement of the device would be broader such that it can measure the loads as high as 7000 lbs.

**Table 1.** Physical properties of the sensor

Thickness	0.208 mm (0.008 in.)
Length	56.8 mm (2.24 in.)
Width	31.8 mm (1.25 in.)
Diameter of sensing area	25.4 mm (1 in.)
Connector	2-pin male square pin
Substrate	Polyester (ex: Mylar)
Pin spacing	2.54 mm (0.1 in.)

Quantitative variables were reported as means and standard deviations, while qualitative variables were reported as numbers and percentages. Generalized estimating equation (GEE) with an exchangeable matrix and linear model was used to assess the correlation between the variables and bite force. Statistical analyses were performed by using SPSS 18 software program (IBM Co., Chicago, IL, USA). P<0.05 was considered statistically significant.

## RESULTS

The mean age of the children was 4.67±0.94 years. There were 30 males (62.26%) and 19 females (38.8%). The mean height of the children was 107.78±12.87 cm (ranging from 62 to 128 cm). The mean weight of the children was 24.29±12.14 kg (ranging from 13 to 52 kg). The mean body mass index (BMI) of the children was 20.73±8.81 kg/m^2^ (ranging from 12.21 to 41.32 kg/m^2^). Twenty-two subjects (44.9%) had a mesial step occlusion, seven subjects (14.3%) had a distal step occlusion, and 20 subjects (40.8%) had a flush terminal plane. Twenty children had extracted teeth; out of which, 9 teeth were in the maxilla (6 on the left side and 3 on the right side) and 11 teeth were in the mandible (5 on the right side and 6 on the left side). None of the children showed symptoms of TMD. Table 2 shows the correlation between the qualitative variables and bite force.

**Table 2.** Descriptive specification of bite force in different subgroups

**Variables**		**Bite force (N)**				

		**Mean**	**Median**	**Minimum**	**Maximum**	**Standard Deviation**
Gender	Male	353.1	352	173	563	88.53
	Female	293.18	285	103	492	103.19
Occlusion type	Mesial step	336.36	345	103	519	80.92
	Distal step	354.57	374	141	473	103.29
	Flush terminal plane	314.08	286.5	121	563	113.15
Side of Jaw	Left	321.31	312	103	492	90.72
	Right	338.43	342	121	563	105.84
Extraction	No	337.91	332	103	563	102.73
	Yes	298.5	298	121	426	73.46

With regard to the correlation between the bite force and quantitative variables, the following results were obtained:

### The side of tooth extraction:

The mean bite force was significantly lower on the side of tooth extraction compared to the contralateral side with a mean difference of 59.64±11.23 N (P<0.001).

### The side of load application:

The bite force on the right side of the jaw was higher than that on the left side by an average of 17.12±9.45 N, but this difference was not significant (P=0.07).

### Gender:

The bite force in males was significantly higher than that in females by 59.9±14.34 N (P<0.001).

### Type of occlusion:

The type of occlusion had no significant effect on the bite force (P>0.05).

### Age:

Increase in age was significantly correlated with the bite force, and by each one-year increase in age, the bite force averagely increased by 30 N (P=0.005).

### Height:

By an increase in height, the bite force significantly increased such that per each 1-cm increase in height, the bite force increased by 3.18 N (P<0.001).

### Weight:

An increase in weight increased the bite force such that each 1-kg increase in weight increased the bite force by 1.34 N, but this correlation was not statistically significant (P=0.7).

## DISCUSSION

Bite force depends on many anatomical and physiological factors. In this study, we assessed the bite force of 3-6-year-old children in primary dentition period with and without unilaterally extracted Ds. The correlations between the bite force and height, weight, gender, type of occlusion and TMD were evaluated. The results showed that the bite force on the side of extraction was significantly lower than that on the contralateral side (P<0.001). Also, the bite force was significantly correlated with height (P<0.001), gender (P<0.001) and age (P=0.005), but the correlations between the bite force and weight (P=7), type of occlusion (P>0.05) and side of the jaw (P=0.07) were not significant. The previous studies on this topic have reported variable bite forces, which are attributed to many anatomical and physiologic factors. The type of occlusion is one of the factors that can affect the bite force. However, in the present study, no association was noted between the bite force and type of occlusion. Rentes et al [17] measured the bite force in three groups of patients with normal occlusion, crossbite, and open bite in primary dentition. They showed that the type of occlusion had no effect on the bite force, and found no significant difference among the three groups [17].

Their finding was in agreement with ours. With regard to the effect of gender, our study showed that boys had a significantly stronger bite force than girls, which was in line with the results of similar previous studies [8,18–22]. The difference in the bite force between males and females is due to the higher muscle strength of males [23] and anatomical differences between males and females [1]. The masseter muscles in males have two types of fibers with a greater diameter than that in females. Hormonal differences also result in stronger muscles in males [24]. Also, females have a lower pain threshold than males, and this could also contribute to a lower bite force in females [25]. After puberty, the maximum bite force in males increases faster than that in females [26].

Ferrario et al [18] reported a greater bite force in males and attributed it to the larger sizes of teeth and periodontal attachment surfaces, which create a stronger force. Palinkas et al [27] evaluated the effect of gender on the bite force and showed that the bite force in males was 30% higher than that in females. However, Abu Alhaija et al [28] found no significant difference in the bite force between males and females, which is in consensus with this survey. Sghaireen et al [11] examined the maximum bite force in primary and permanent teeth and assessed the correlation of the maximum bite force with BMI and gender. The result was that the maximum bite force was higher at the site of first permanent molars (in comparison with primary molars), and the bite force exerted by primary second molars was significantly correlated with gender. Also, the bite force applied by first permanent molars was significantly correlated with gender, age and the bite force of second primary molars [11].

The present study showed no significant difference in the bite force on the right and left sides of the jaw. The following explanation might justify this finding: first, chewing with one side of the jaw could be habitual, and factors such as occlusal interferences or pain of a carious tooth could play a role in the patient’s preference for the dominant side of chewing. Also, the right and left sides of the jaw and the upper and lower jaws are related, and thus, even by chewing with one side of the jaw, the muscles on both sides are involved.

The results of this study showed that the bite force was significantly correlated with age and height. However, the association between the weight and bite force was not significant. An increase in the height significantly increased the bite force. Weight gain in children is mainly due to fat deposition rather than an increase in the muscle mass [2], which can be an explanation for no significant association between the bite force and weight in the present study. Pereira et al [29] evaluated the correlation of the bite force at the molar site with the age, height, and weight of 6-8-year olds, and found that the age, height and weight were significantly correlated with the bite force. Their results regarding the correlation of the age and height with the bite force were in line with the findings of our study, but not in terms of the correlation with the weight. Palinkas et al [27] examined the effect of age on the bite force in patients between the ages of 7 and 80 years and showed that the bite force increases with an increase in age and the thickness of masticatory muscles, but in old age, the bite force decreases due to muscle atrophy and a decrease in the muscle thickness. The same result was found in our study in children.

Our study showed a significantly lower bite force on the extraction side, which can be due to a fewer number of teeth on the respective side of the jaw. Also, children normally chew with the side with no extracted teeth, and this leads to further strengthening of the muscles on the mentioned side of the jaw.

One of the limitations of this study was the fact that by using a sensor, only the magnitude of bite force can be measured, but the actual chewing patterns of the individuals cannot be determined, and the patient might not be able to properly simulate normal chewing when biting on the sensor. Thus, future studies are required to find the actual chewing patterns of patients and to simulate them when measuring the bite force to obtain more accurate results. Future studies with larger sample sizes and on patients in mixed dentition period are required to better elucidate this topic.

## CONCLUSION

The data of the present study revealed that unilateral extraction of a primary first molar in primary dentition phase decreases the bite force on the respective side of the jaw. The bite force is significantly correlated with height, gender, and age, but not with weight, type of occlusion or side of the jaw.
